# Predicting the Outcome of Lymphovenous Anastomosis for Lower Extremity Lymphedema through Lymphoscintigraphy

**DOI:** 10.7150/ijms.111506

**Published:** 2025-06-23

**Authors:** Eun Ji Han, Ji Won Moon, Ji Min Son, Mihai Oltean, Mats Hellström, Francesco Boccardo, Min Jong Song

**Affiliations:** 1 Division of Nuclear Medicine, Department of Radiology, College of Medicine, The Catholic University of Korea, Seoul, Republic of Korea.; 2 Department of Obstetrics and Gynecology, College of Medicine, The Catholic University of Korea, Seoul, Republic of Korea.; 3 Department of Transplantation Surgery, Sahlgrenska Academy, University of Göthenburg, Göteborg, Sweden.; 4 Department of Obstetrics and Gynecology, Sahlgrenska Academy, University of Göthenburg, Göteborg, Sweden.; 5 Laboratory for Transplantation and Regenerative Medicine, Sahlgrenska Academy, University of Göthenburg, Göteborg, Sweden.; 6 Unit of Lymphatic Surgery, Department of Surgery, IRCCS S. Martino Hospital - IST, National Cancer Institute for Cancer Research, University of Genoa, Genova, Italy.

**Keywords:** lymphedema, lymphoscintigraphy, lymphovenous anastomosis, outcome, transport index

## Abstract

**Background:** Lymphovenous anastomosis (LVA) is an effective treatment for restoring lymphatic function in patients with lymphedema. This study aimed to assess the predictive value of preoperative lymphoscintigraphy in female patients undergoing LVA for lower extremity lymphedema (LEL).

**Methods:** Female patients with unilateral LEL who underwent preoperative lymphoscintigraphy followed by LVA were retrospectively examined. In the lymphoscintigraphy, the transport index (TI) was calculated based on five visual interpretation criteria: lymphatic transport kinetics, dermal backflow pattern, time to appearance of lymph nodes, visualization of lymph nodes, and visualization of vessels. For volume assessment, the LEL index (LELI) was calculated as the sum of circumferences at five predefined sites of the lower extremity, divided by body mass index. LELI was measured before and after LVA at 1, 3, and 6 months. Postoperative changes in LELI were compared with preoperative variables, including TI.

**Results:** The study included 45 female patients (mean age 56 ± 10 years) with unilateral LEL, of whom 78% had clinical stage 3 lymphedema. The mean TI of the affected lower extremities at 240 and 120 min was 25.5 ± 11.0 and 26.5 ± 11.1, respectively. TI was significantly associated with clinical stage and preoperative volume excess. Postoperatively, the mean LELI reduction was 7 ± 5% at 1 month, 8 ± 5% at 3 months, and 6 ± 7% at 6 months. Significant negative correlations were found between the TI at both 240 and 120 min and postoperative LELI changes at 3 and 6 months (p < 0.05).

**Conclusions:** Preoperative lymphoscintigraphy, specifically the TI, is valuable for assessing the severity of lymphedema and predicting short-term outcomes of LVA in female patients with LEL. The TI can be calculated from lymphoscintigraphy performed up to 2 h.

## Introduction

Lymphedema is a chronic, debilitating condition characterized by extremity swelling due to impaired lymphatic drainage. It can be classified as primary or secondary, depending on the underlying cause. Secondary lymphedema results from damage to the lymphatic system caused by disease, trauma, lymphadenectomy, and/or radiation therapy and is most commonly seen after the treatment of malignancy in developed countries 1. Secondary lower extremity lymphedema (LEL) is a common health issue in female patients treated for gynecological cancer. While the incidence varies widely across different cancer types (1-81%), approximately half of female patients treated for gynecological cancer develop LEL, and 60% of these cases persist 2.

LEL is a progressive disease that worsens over time without proper management, leading to increased physical disability, including pain, heaviness, tightness, recurrent infections, elephantine skin changes, and reduced quality of life 3, 4. Unfortunately, no curative treatment has been established for LEL. Complex physical decongestive therapy (CPDT), which includes manual lymphatic drainage, compression garments, exercise, and skin care, is a commonly used conservative treatment to prevent LEL progression and alleviate symptoms 5, 6. However, patients receiving conservative treatment must manage the condition for life, and most advanced cases of LEL are resistant to this approach. Consequently, surgery has been considered an effective treatment option. Liposuction, a reductive surgery, can be performed for advanced LEL and leads to remarkable direct volume reduction 7. Recently, microsurgical physiological interventions aimed at restoring lymphatic circulation have been developed to achieve more fundamental improvements. Lymphovenous anastomosis (LVA) is a physiological technique that anastomoses functioning lymphatic vessels to subdermal venules, allowing excess lymphatic fluid to drain directly into the venous system 8, 9.

Lymphoscintigraphy is a simple and minimally invasive method for assessing the functional status of the lymphatic system. It is considered the gold standard method for imaging-based diagnosis of lymphedema, allowing for the evaluation of lymphatic drainage and lymphedema severity. In addition, it has recently been used to evaluate treatment response and predict prognosis in patients with lymphedema 10, 11. Lymphoscintigraphic findings can be analyzed qualitatively or quantitatively. Qualitative analysis is easy to apply in clinical practice and plays a key role in interpreting lymphoscintigraphy. However, this is not sufficient for classifying the severity of individual cases or evaluating the outcomes of surgical interventions 12. Although several methods for quantitative analysis have been proposed to provide a more detailed and objective assessment, they have not been widely adopted in clinical practice owing to technical inconvenience and inter-hospital variability 10, 13. A standardized protocol and reading criteria for lymphoscintigraphy have not yet been established, and discrepancies have been noted in the choice of radiopharmaceuticals, injection sites and types, stress application, and imaging acquisition methods among different hospitals 14. Several radiopharmaceuticals, such as ^99m^Tc-antimony trisulfide colloid, ^99m^Tc-tin colloid, ^99m^Tc-human serum albumin, and ^99m^Tc-phytate, can be used for lymphoscintigraphy; however, few studies have directly compared their performances 15-17.

Therefore, this study aimed to evaluate the clinical utility of preoperative lymphoscintigraphy in female patients with LEL who underwent LVA and assess whether it predicts short-term postoperative outcomes using several qualitative parameters and a derived formula.

## Materials and Methods

### Patient population

This retrospective study included female patients who underwent lymphoscintigraphy and received LVA for LEL between November 2017 and July 2019 at the Department of Gynecology of our hospital. Clinical variables, including age, history of LEL, body mass index (BMI), and clinical stage (Table 1) 18, were obtained from medical records. The exclusion criteria were definite bilateral LEL or loss to follow-up with unknown postoperative outcomes. All patients underwent ultrasonography to evaluate the veins in the lower extremity (LE), and no evidence of deep vein thrombosis was found.

This study was approved by the Institutional Review Board of Catholic Medical Center (IRB No. DC19RISI0111). The Institutional Ethics Committee waived the need for patient consent for this retrospective review of imaging studies and clinical data.

### Lymphoscintigraphy

^99m^Tc-phytate (74 MBq) was injected subcutaneously into the first and fourth interdigital spaces of both feet using a 27-gauge needle. Patients were immediately encouraged to perform repeated gripping exercises for 10 min to facilitate the transport of radiopharmaceuticals. Studies were conducted using a dual-headed gamma camera (E-CAM; Siemens Medical Solutions, Erlangen, Germany). Anterior and posterior whole-body images were acquired at 15, 30, 60, 120, 180, and 240 min after injection. Several qualitative parameters were binary assessed for each LE: (1) lymphatic transport kinetics (K) (no or mild delay versus marked delay or missing transport); (2) dermal backflow (DBF) (D) (normal versus DBF or transport stop); (3) time to appearance of lymph nodes (LNs) (T) (≤ 60 min versus > 60 min); (4) visualization of LNs (N) (visualization versus no visualization); (5) visualization of the main lymphatic vessel (V) (visualization versus no visualization); and (6) visualization of collateral flow (C) (no visualization versus visualization). In addition, the transport index (TI) at 240 min (TI_240_) was calculated by scoring each parameter using the following formula previously proposed (Table 2) 12:

TI = K + D + T*0.04 + N + V

Additionally, the TI at 120 min (TI_120_) was calculated using the 15-, 60-, and 120-min images. The TI ranged from 0.6 to 45. All lymphoscintigraphy findings were reviewed twice by a nuclear medicine physician who was blinded to the clinical information. The intra-class coefficient between the first and second readings was 0.983. In case of disagreement between the first and second readings, a consensus was reached with another nuclear medicine physician.

### Evaluation of postoperative outcomes

The circumference of the LE was measured using a tape measure at five predefined sites: (1) the superior edge of the patella, (2) 10 cm above and (3) below the patella, (4) the lateral malleolus, and (5) the dorsum of the foot. All measurements were performed manually by the same gynecologist. The LEL index (LELI) was calculated by dividing the sum of the squares of the circumferences at the five predefined sites of the LE by the BMI for comparison across different patients 19, 20. The LELI was measured before and after LVA at 1, 3, and 6 months. The changes in LELI (%ΔLELI) after LVA at 1, 3, and 6 months were then calculated to assess the outcomes of LVA.

### Statistical analysis

Categorical variables are presented as absolute numbers and percentages, while continuous variables are expressed as mean ± standard deviation (SD) and range. The Wilcoxon signed-rank test was used to compare two related variables. The Mann-Whitney test was used for comparison between two groups and Spearman correlation analysis was used to assess the association between two continuous variables. All statistical analyses were conducted using the Statistical Package for Social Sciences version 26.0 (IBM Corp., Armonk, NY, USA). Differences were considered statistically significant at a p value of < 0.05.

## Results

### Patients

A total of 59 female patients underwent preoperative lymphoscintigraphy followed by LVA, of which 9 had bilateral LEL and 5 were lost to postoperative follow-up. Consequently, 45 patients were included in the final analysis. Most patients had previously received treatment for gynecological cancers, and only one patient had primary lymphedema. According to the clinical staging system, the majority (78%) of patients had stage 3 lymphedema. In the preoperative volume measurements, the LELI of the affected LEs was significantly higher than that of the unaffected contralateral LEs (p < 0.001). Patient characteristics are provided in Table 3.

### Assessments of preoperative lymphoscintigraphy

Qualitative assessments of the affected LEs in the 45 lymphoscintigraphies are presented in Table 4. The mean TI_240_ of the affected LEs was 25.5 ± 11.0 (range, 6.6-45). Among the 45 unaffected contralateral LEs, 4 exhibited mildly elevated TI (4.2, 6.6, 7.2, and 8.4). When analyzing only the 15-, 60-, and 120-min images, the mean TI_120_ of the affected LEs was 26.5 ± 11.1 (range, 6.6-45). Of the 45 affected LEs, 31 (69%) had identical TI_240_ and TI_120_, while 14 (31%) showed a slightly higher TI_120_ than TI_240_ (mean, 3.2 ± 1.7; range, 1.2-5.8). The mean TI_240_ increased gradually with clinical stage (13.8 ± 2.5 in stage 2; 26.4 ±10.6 in stage 3; 31.2 ± 12.5 in stage 4), and the differences were significant (p = 0.029). The mean TI_120_ also showed a similar trend (p = 0.026). Both TI_240_ and TI_120_ exhibited moderate positive correlations with the volume difference between the affected and unaffected contralateral LEs (r = 0.334, p = 0.024 for TI_240_; r = 0.323, p = 0.03 for TI_120_).

### Postoperative evaluation

In postoperative volume assessments, the mean LELI of the affected LEs was 218.4 ± 56.8 (range, 170-275.3) at 1 month, 175.7 ± 98.7% (range, 156.5-290) at 3 months, and 147.9 ± 110.2% (range, 147.9-292.3) at 6 months. The mean %ΔLELI was 6.5 ± 4.9% (range, -1.8-26.6) at 1 month, 7.9 ± 5% (range, 1.3-17.1) at 3 months, and 5.6 ± 6.8% (range, -21.5-18.1) at 6 months. A moderate positive linear relationship was observed for %ΔLELI between 1 and 3 months (r = 0.464, p = 0.003) and a strong positive linear relationship was found for %ΔLELI between 3 and 6 months (r = 0.732, p < 0.001).

One month postoperatively, only one patient exhibited a 1.8% increase in LELI; however, reductions of 6% and 4.4% were observed at 3 and 6 months, respectively. Three months postoperatively, all patients showed a reduction in LELI. Six months postoperatively, two patients showed a 2.3% and 21.5% increase in LELI, although their LELI had been reduced at 1 and 3 months.

**Figure 2:**
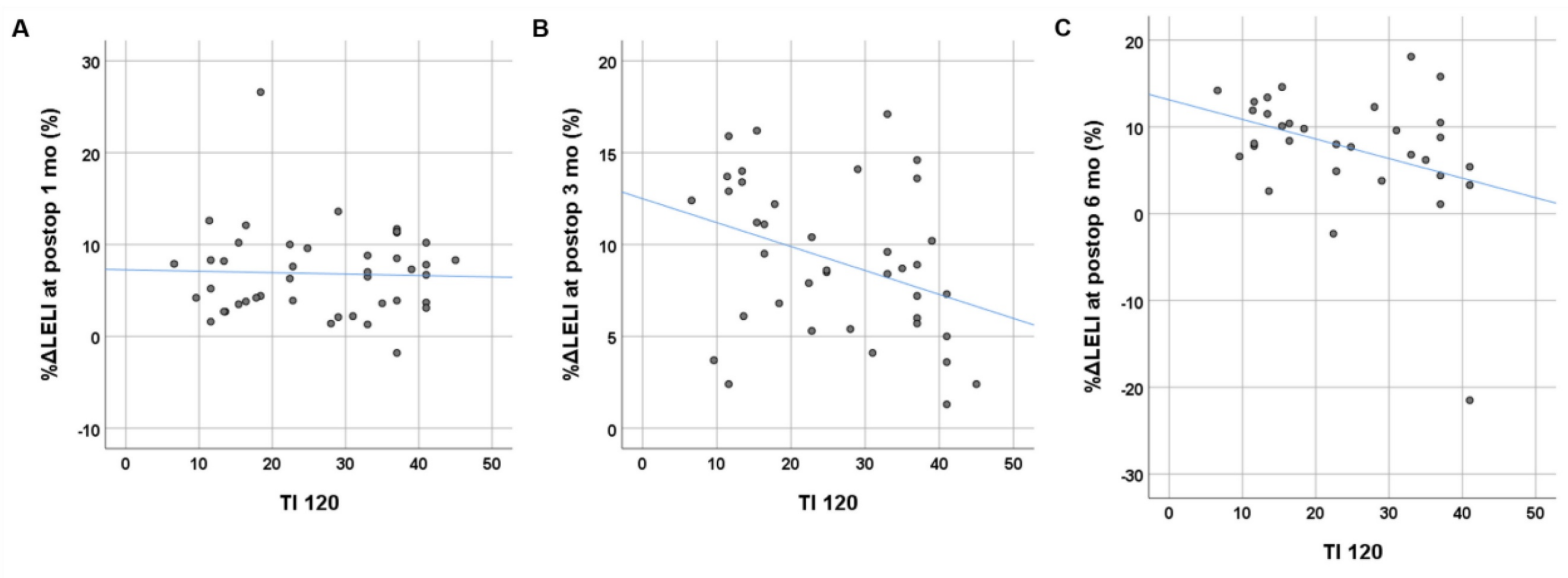
Relationship between the transport index (TI) derived from preoperative lymphoscintigraphy and postoperative changes in lower extremity lymphedema index (%ΔLELI). The TI at 120 min shows a moderate negative linear correlation with %ΔLELI at 3 months (B) and 6 months (C) postoperatively, but no correlation is observed at 1 month postoperatively (A).

Age, duration of LEL, BMI, clinical stage, and preoperative volume excess were not significantly associated with postoperative %ΔLELI at any time point (p > 0.05). Among the qualitative parameters from lymphoscintigraphy, visualization of the main lymphatic vessel was statistically associated with %ΔLELI at 3 months (p = 0.045), and lymphatic transport kinetics was statistically associated with %ΔLELI at 6 months (p = 0.03) (Table 5). Both TI_240_ and TI_120_ showed significant negative correlations with postoperative %ΔLELI at 3 months (r = -0.332, p = 0.039 for TI_240_; r = -0.366, p = 0.022 for TI_120_) and 6 months (r = -0.352, p = 0.048 for TI_240_; r = -0.370, p = 0.037 for TI_120_), but not with %ΔLELI at 1 month (p = 0.739 for TI_240_; p = 0.846 for TI_120_) (Figure 2).

## Discussion

In the present study, we assessed the clinical utility of preoperative lymphoscintigraphy using several qualitative parameters and a derived formula in female patients with LEL who underwent LVA. The TI derived from lymphoscintigraphy was significantly associated with the preoperative stage and the excess volume of the affected LE. Additionally, the TI showed a negative correlation with postoperative volume reduction rates at 3 and 6 months. The TI at 120 min demonstrated similar results to that at 240 min.

Early and accurate diagnosis and evaluation of LEL are crucial for determining the appropriate timing for conservative treatment and identifying candidates for surgery 21. While the International Society of Lymphology classification is the most commonly used staging system for peripheral lymphedema, the three-point scale is too simplistic and inadequate for determining surgical candidates and predicting postoperative outcomes 22, 23. To address this, we further classified the LEL severity into five categories using the Campisi clinical staging system 18. Despite using a more detailed classification, most patients were diagnosed at a specific stage (clinical stage 3). For a more accurate evaluation of LEL, an additional tool to complement the clinical staging system is necessary. In our study, the TI derived from lymphoscintigraphy positively correlated with the preoperative excess volume of the affected LE. Moreover, lymphoscintigraphy can simultaneously assess bilateral LEL. Although our study included only patients with unilateral LEL, four patients unexpectedly showed mildly elevated TI (range, 4.2-8.4) in the unaffected contralateral LE. This suggests mild lymphatic impairment in early lymphedema, as cancer-related LEL is often bilateral 1.

Previous studies have reported promising results for microsurgical physiological reconstructions including LVA, lympho-lymphatic bypass, and vascularized LN transfer for peripheral lymphedema 24. A systemic meta-analysis of 27 studies showed that microsurgical interventions for peripheral lymphedema resulted in both objective and subjective improvements, with a mean follow-up of 3.3 years postoperatively, despite differences in patient selection, surgical technique, and assessment of postoperative outcomes 25. After interventions, the excess circumference and volume were reduced by 49% and 57%, respectively. In subgroup analyses, the reduction in excess circumference was greater in the LEL group than in the upper extremity lymphedema group (57% vs. 46%). Subjective assessment revealed that 91% of patients reported subjective improvements, and only 11% did not experience any improvement. A recent meta-analysis of 74 studies on LVA outcomes for LEL reported objective improvement rates of 23-100% 26. In our study, most patients with LEL showed volume reduction after LVA that persisted for 6 months. The volume reduction rate was the highest 3 months postoperatively, with a slight decline 6 months postoperatively (mean LELI reduction: 8% at 3 months → 6% at 6 months). A longer follow-up period is needed to evaluate the long-term patency of LVA.

CPDT is the most common treatment for peripheral lymphedema, with the severity of the condition being the primary predictor of treatment response 27, 28. Several studies have attempted to predict the response to CPDT using lymphoscintigraphy. Lymphatic dysfunction on lymphoscintigraphy is characterized by delayed or absent visualization of the main lymphatic vessels and/or regional LNs, the presence of DBF, and collateral lymphatic channels 12. Previous studies have shown that visualization of inguinal or axillary LNs on lymphoscintigraphy predicts a positive response to CPDT in patients with peripheral lymphedema 29, 30. Another study suggested that the presence of main lymphatic vessels without collateral flow on lymphoscintigraphy was the best predictor of success 31. A study involving 152 patients with gynecological cancer-related LEL found that the severity of DBF on lymphoscintigraphy was significantly associated with the response to CPDT 32.

Previous studies have also indicated that lymphoscintigraphy could help predict outcomes following microsurgical physiological interventions in patients with peripheral lymphedema. Two studies have specifically reported that DBF on lymphoscintigraphy significantly predicts the success of LVA in patients with LEL 33, 34. In this study, no statistically significant difference was observed in %ΔLELI at any time point between normal cases and those with DBF or transport stop. In the subgroup analysis, statistically significant differences were observed in %ΔLELI at 3 months between normal cases and those with partial DBF (p = 0.046); between cases with partial DBF and those with diffuse DBF (p = 0.046); and between cases with diffuse DBF and those with transport stop (p = 0.04). These partially significant differences may be attributed to the small sample size, highlighting the need for further studies with larger cohorts. Lymphedema manifests clinically in a variety of locations, symptoms, and severities and is represented by different lymphoscintigraphic findings. More detailed and complex classification systems for lymphoscintigraphy have been proposed to better capture the pathogenic mechanisms of lymphedema and improve the prediction of microsurgical intervention outcomes 12, 35, 36. The previously proposed TI is a continuous variable that comprehensively reflects the condition of the main lymphatic vessels, regional LNs, and DBF, showing good predictive performance for the success of lymphatic vessel transplantation 12. In the present study, we applied the TI to patients undergoing LVA for LEL. Our results showed a significant negative correlation between the TI derived from preoperative lymphoscintigraphy and the volume reduction rates at 3 and 6 months postoperatively. Previous studies have also reported that patients with early-stage lymphedema tend to have better outcomes after LVA than those with advanced-stage lymphedema 26, 37, 38. Additionally, prophylactic LVA has been shown to prevent lymphedema in patients undergoing lymphadenectomy 39, 40. Most patients in our study had advanced diseases (clinical stage 3). In the subgroup analysis of 35 patients with clinical stage 3 lymphedema, the results were similar to the overall analysis: TI_120_ showed a significantly negative correlation with postoperative %ΔLELI at 3 months (r = -0.406, p = 0.026) and 6 months (r = -0.417, p = 0.034), but not at 1 month (p = 0.866). These findings suggest that TI derived from lymphoscintigraphy could serve as a complementary tool for assessing the severity of LEL and predicting LVA outcomes, enabling more individualized treatment plans.

The use of the TI is limited by the long acquisition time of up to 4 h. We acquired both TI_240_ and TI_120_ on lymphoscintigraphy, and 10 (22%) of the 45 patients showed a slight increase from TI_240_ to TI_120_. Although TI_120_ was higher than TI_240_, both TI_240_ and TI_120_ were significantly associated with preoperative excess volume and postoperative volume reduction rates. Therefore, using TI_120_ instead of TI_240_ may reduce patient discomfort due to shorter acquisition times and improve the operational efficiency of the scanner.

This study has some limitations, including a small sample size, its retrospective design, and a short follow-up period. Long-term outcomes of microsurgical physiological interventions depend not only on preoperative conditions but also on patient's continued compliance with postoperative CPDT 23. Further prospective studies with larger cohorts and longer follow-up periods are warranted to confirm these findings.

## Conclusions

TI derived from preoperative lymphoscintigraphy is effective for assessing the severity of LEL and predicting short-term outcomes of LVA in female patients. Lymphoscintigraphy acquisition for up to 2 h is sufficient for calculating the TI.

### Ethics committee approval and patient consent

This study was approved by the Institutional Review Board of Catholic Medical Center (IRB No. DC19RISI0111). The Institutional Ethics Committee waived the need for patient consent for this retrospective review of imaging studies and clinical data.

### Data availability

The data presented in this study are available upon reasonable request. Data cannot be directly shared on public repositories due to the national personal data protection act.

## Figures and Tables

**Figure 1:**
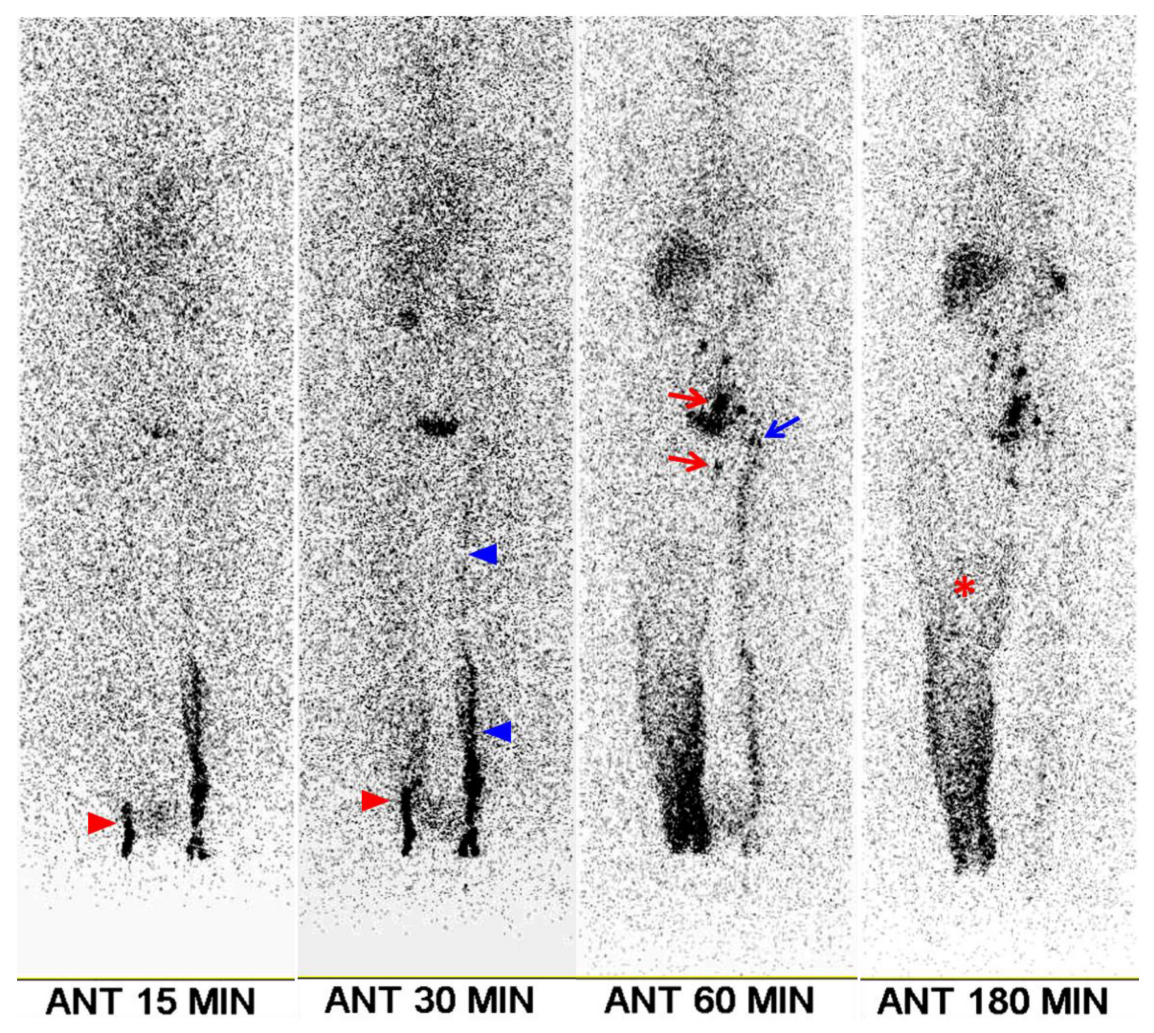
A 70-year-old female patient with right leg edema lasting 4 months. She had undergone surgery, chemotherapy, and radiotherapy for ovarian cancer 6 years prior. Clinical examination revealed stage 2 lymphedema. Lymphoscintigraphy shows minimal visualization of lymphatic flow in the right leg (red arrowheads), along with diffuse dermal backflow (asterisk). Right ilio-inguinal nodes are visible starting at the 60-min image (red arrows). The left leg exhibits mildly delayed lymphatic flow (blue arrowheads), with mild visualization of the left ilio-inguinal nodes from the 60-min image (blue arrow). The transport index was calculated as 15.4 for the right leg and 8.4 for the left leg. Following lymphovenous anastomosis, the lower extremity lymphedema index of the right leg decreased by 10.2% at 1 month, 16.2% at 3 months, and 14.6% at 6 months.

**Table 1:** Campisi clinical staging system for peripheral lymphedema

Stage 1a	No edema
Stage 1b	Mild edema, reversible with declivous position and night rest
Stage 2	Persistent edema that regresses only partially with declivous position and night rest
Stage 3	Persistent edema that continually becomes more severe (recurrent acute erysipeloid lymphangitis)
Stage 4	Fibrotic lymphedema (with initial lymphostatic warts) and column-shaped limb
Stage 5	Elephantiasis with severe limb deformation, scleroindurative pachydermitis, and widespread lymphostatic warts

**Table 2:** Qualitative interpretation scoring for the TI

	Score 0	Score 3	Score 5	Score 9
Lymphatic transport kinetics (K)	No delay	Mild delay	Marked delay	Missing transport
DBF (D)	Normal	Partial	Diffuse	Transport stop
Time to appearance of LNs (T)	Time in min to the first appearance of ilio-inguinal LNs			No visualization
Visualization of LNs (N)	Clearly visible	Mildly visible	Hardly visible	No visualization
Visualization of main lymphatic vessel (V)	Clearly visible	Mildly visible	Hardly visible	No visualization

TI, transport index; DBF, dermal backflow; LN, lymph node

**Table 3:** Patient characteristics

Characteristic		No. (%)
Age (years)	Mean ± SD (range)	56 ± 10 (25-78)
Affected side	Right	18 (40%)
	Left	27 (60%)
Duration of lymphedema (years)	Mean ± SD (range)	6.4 ± 5.8 (0.3-21.0)
History of lymphangitis	Yes	18 (40%)
	No	27 (60%)
Primary cancer site	Cervix	32 (71%)
	Ovary	8 (18%)
	Endometrium	4 (9%)
	No malignancy	1 (2%)
Cancer-related treatment	Pelvic lymphadenectomy only	26 (58%)
	Pelvic lymphadenectomy and radiotherapy	18 (40%)
	No treatment	1 (2%)
BMI (kg/m^2^)	Mean ± SD (range)	25.3 ± 3.5 (19.1-33.0)
Campisi clinical stage	2	5 (11%)
	3	35 (78%)
	4	5 (11%)
LELI	Affected LE (mean ± SD [range])	251.3 ± 28.4 (176.3-298.8)
	Unaffected LE (mean ± SD [range])	200.2 ± 18.8 (159.8-238.9)

BMI, body mass index; SD, standard deviation; LE, lower extremity; LELI, lower extremity lymphedema index

**Table 4:** Number of cases based on qualitative parameters from lymphoscintigraphy

	Score 0	Score 3	Score 5	Score 9
Lymphatic transport kinetics (K)	1	16	16	12
DBF (D)	2	7	34	2
Time to appearance of LNs (T)	15 min (n=6); 30 min (n=5); 60 min (n=6); 120 min (n=5); 180 min (n=2)			21
Visualization of LNs (N)	8	6	8	23
Visualization of main lymphatic vessel (V)	3	13	22	7
Visualization of collateral flow (C)	No visualization (n=19)			26

DBF, dermal backflow; LN, lymph node

**Table 5:** Relationships between qualitative parameters from lymphoscintigraphy and postoperative volume change

	Postoperative %ΔLELI (p value)		
	At 1 month	At 3 months	At 6 months
Lymphatic transport kinetics	0.920	0.058	0.03*
DBF	0.158	0.098	0.755
Time to appearance of LNs	0.628	0.109	0.109
Visualization of LNs	0.453	0.368	0.226
Visualization of main lymphatic vessel	0.745	0.045*	0.169
Visualization of collateral flow	0.115	0.160	0.985

%ΔLELI, change in lower extremity lymphedema index; DBF, dermal backflow; LN, lymph node
